# Genomic insights into the evolution of secondary metabolism of *Escovopsis* and its allies, specialized fungal symbionts of fungus-farming ants

**DOI:** 10.1128/msystems.00576-24

**Published:** 2024-06-21

**Authors:** Aileen Berasategui, Hassan Salem, Abraham G. Moller, Yuliana Christopher, Quimi Vidaurre Montoya, Caitlin Conn, Timothy D. Read, Andre Rodrigues, Nadine Ziemert, Nicole Gerardo

**Affiliations:** 1 Department of Biology, Emory University, Atlanta, Georgia, USA; 2 Cluster of Excellence-Controlling Microbes to Fight Infections, University of Tübingen, Tübingen, Germany; 3 Mutualisms Research Group, Max Planck Institute for Biology, Tübingen, Germany; 4 Amsterdam Institute for Life and Environment, Vrije Universiteit Amsterdam, Amsterdam, the Netherlands; 5 Division of Infectious Diseases, Emory University School of Medicine, Atlanta, Georgia, USA; 6 Instituto de Investigaciones Científicas y Servicios de Alta Tecnología, Ciudad del Saber, Panamá City, Panama; 7 Department of General and Applied Biology, São Paulo State University (UNESP), Institute of Biosciences, Rio Claro, São Paulo, Brazil; 8 Department of Biology, Berry College, Mount Berry, Georgia, USA; 9 Translational Genome Mining for Natural Products, Interfaculty Institute of Microbiology and Infection Medicine Tübingen (IMIT), Interfaculty Institute for Biomedical Informatics (IBMI), University of Tübingen, Tübingen, Germany; University of Connecticut, Storrs, Connecticut, USA

**Keywords:** secondary metabolism, symbiosis, parasitism

## Abstract

The metabolic intimacy of symbiosis often demands the work of specialists. Natural products and defensive secondary metabolites can drive specificity by ensuring infection and propagation across host generations. But in contrast to bacteria, little is known about the diversity and distribution of natural product biosynthetic pathways among fungi and how they evolve to facilitate symbiosis and adaptation to their host environment. In this study, we define the secondary metabolism of *Escovopsis* and closely related genera, symbionts in the gardens of fungus-farming ants. We ask how the gain and loss of various biosynthetic pathways correspond to divergent lifestyles. Long-read sequencing allowed us to define the chromosomal features of representative *Escovopsis* strains, revealing highly reduced genomes composed of seven to eight chromosomes. The genomes are highly syntenic with macrosynteny decreasing with increasing phylogenetic distance, while maintaining a high degree of mesosynteny. An ancestral state reconstruction analysis of biosynthetic pathways revealed that, while many secondary metabolites are shared with non-ant-associated *Sordariomycetes*, 56 pathways are unique to the symbiotic genera. Reflecting adaptation to diverging ant agricultural systems, we observe that the stepwise acquisition of these pathways mirrors the ecological radiations of attine ants and the dynamic recruitment and replacement of their fungal cultivars. As different clades encode characteristic combinations of biosynthetic gene clusters, these delineating profiles provide important insights into the possible mechanisms underlying specificity between these symbionts and their fungal hosts. Collectively, our findings shed light on the evolutionary dynamic nature of secondary metabolism in *Escovopsis* and its allies, reflecting adaptation of the symbionts to an ancient agricultural system.

**IMPORTANCE:**

Microbial symbionts interact with their hosts and competitors through a remarkable array of secondary metabolites and natural products. Here, we highlight the highly streamlined genomic features of attine-associated fungal symbionts. The genomes of *Escovopsis* species, as well as species from other symbiont genera, many of which are common with the gardens of fungus-growing ants, are defined by seven chromosomes. Despite a high degree of metabolic conservation, we observe some variation in the symbionts’ potential to produce secondary metabolites. As the phylogenetic distribution of the encoding biosynthetic gene clusters coincides with attine transitions in agricultural systems, we highlight the likely role of these metabolites in mediating adaptation by a group of highly specialized symbionts.

## INTRODUCTION

Most symbionts are specialists. At broad scales, most symbionts can associate with some host species and not others. At finer scales, many strains may be specialized on particular host genotypes within a species (1). While host range is constrained by different evolutionary processes, including tradeoffs and coevolutionary dynamics (2, 3), the molecular mechanisms underlying specialization and the evolutionary ecology of specificity have yet to be clearly linked. Similarly, little is known about the genomic architecture underlying the evolution of symbiont specialization, the genomic consequences of host shifts, and the genetic basis of shifts along the parasitism to mutualism continuum that underlies most symbioses.

Fungal symbionts are genetically tractable models for the study of host fidelity due to their diverse lifestyles and the occurrence of very closely related species that differ from each other primarily in their host range (4). Secondary metabolites, small molecules that are not necessary for the growth of an organism but aid in survival, play essential roles during fungal infection (5) and are known to affect the niche breath of fungal pathogens (4, 6, 7). Typically, specialists harbor a contracted array of specialized metabolites relative to generalists (4), reflecting the metabolic constraints that they experience in attempting to exploit different hosts. However, this is not always the case. Broad host range mutualists such as mycorrhizal fungi, associated with most land plants, have a limited ability to produce toxins (8). This likely reflects their biotrophic lifestyle, where the production of toxins may compromise the survival of their host, which they require alive (9, 10). Given their role in mediating species interactions, secondary metabolites are central to arms-races dynamics in antagonistic interactions (11, 12). Thus, their origin and distribution can reflect adaptation to specific host environments (7).

*Escovopsis* (*Hypocreales: Hypocreaceae*) is a specialized (13–15), diverse group of fungi found in the gardens of fungus-farming ants (*Hymenoptera: Attini*) (16). Currently, there are 25 described species (17), some of which have been well-studied for their ability to parasitize the ants’ fungal cultivars (13–15). *Escovopsis* strains can be virulent parasites of fungus-growing ant agriculture, causing garden biomass loss and colony decline (16, 18, 19). While it is presumed that most species in the group are similarly virulent, infection by certain species appears to be not as lethal, suggesting that the ecological role and evolutionary implications of these symbionts are not fully understood (20–23). In recognition of their morphological and ecological diversity, a recent study split the *Escovopsis* genus into multiple genera (i.e., *Escovopsis, Luteomyces,* and *Sympodiorosea*) (24). Here, we sometimes refer to all members of the group with the common name escovopsis symbionts for simplicity, restricting the use of *Escovopsis* to those strains within the genus.

Fungus-farming ants are a monophyletic group of obligate agriculturalists (25). Attines feed their cultivated fungi ("cultivars") with plant material, and in turn, the cultivar represents the ants’ primary food source. Different attine lineages practice different modes of agriculture, exhibiting a high degree of specificity toward their cultivars (26, 27), and these different agricultural systems are generally associated with different *Escovopsis, Sympodiorosea,* and *Luteomyces* species (28). The ancestral system, lower agriculture, is practiced by a group of ants that cultivate fungi in the *Agaricales*. While most of the ant species in this system grow their cultivars in the form of mycelium, some ants in the lower agriculture system subsist on *Agaricales* that grow in yeast form, giving rise to the name of yeast agriculture. While *Sympodiorosea* and *Luteomyces* infections of mycelial-growing lower attine ant gardens are common, infection of yeast gardens has never been found (25). The third agricultural system is known as coral agriculture, in which a group of ants within the *Apterostigma* genus exploits fungus in the *Pterulaceae* family. Infections of coral gardens are also common, and include infection by *Escovopsis*, *Luteomyces,* and other related taxa (13, 24). While lower attines, practicing lower, yeast, and coral agriculture, are characterized by providing their cultivars with dead vegetative material, higher attines (practicing generalized higher agriculture and leaf-cutter agriculture) provide their fungal mutualists with freshly cut plant material (25). The two agricultural systems of higher attines are characterized by the obligate lifestyle of the cultivar, which cannot survive without association with the ants. Generalized higher agriculture is practiced by ants cultivating a derived clade of agaricaceous fungi, whereas in the most derived agricultural system, that of leaf-cutter agriculture, a single fungal species *Leucoagaricus gongylophorus* is responsible for ant survival. Higher agriculture gardens are commonly infected with *Escovopsis*, most of which are *Escovopsis* spp. closely related to the best studied species, *Escovopsis weberi* (29).

Escovopsis symbionts show a high degree of host fidelity, being able to infect some cultivars but not others. This degree of partner specificity suggests a long history of coevolution, as demonstrated by the phylogenetic congruence between attines, their cultivars, and escovopsis symbionts, particularly at the broad interspecific scale (13). To manage infections, ants actively weed infected portions of garden, and many attine species associate with actinomycete *Pseudonocardia* that synthesize antifungal compounds that inhibit escovopsis symbiont growth (30).

Despite consistent patterns of co-diversification across the tripartite interaction between the ants, their cultivars, and escovopsis symbionts (13), and the outsized role of natural products in mediating fungal specialization, the secondary metabolism of these microbes remains relatively unexplored relative to the evolutionary ecology of attine ants and their cultivars. Only a few *Escovopsis*-derived compounds have been identified (31, 32), though recent genome annotation indicates the potential to produce many more (33). Here, we performed a combination of long- and short-read genome sequencing, assembly, and annotation to describe the chromosomal architecture, conservation, and organization of escovopsis symbionts, which will facilitate future annotation of the biosynthetic machinery. After defining the secondary metabolism across the group and spanning representative host ranges, we contextualize the distribution of biosynthetic gene clusters relative to patterns of specialization and fidelity. Through comparative genomics, extensive manual curation of biosynthetic gene clusters, and ancestral state reconstruction, we outline a group of symbionts whose secondary metabolism broadly reflects the dynamic patterns of cultivar recruitment and replacement by attine ants.

## MATERIALS AND METHODS

### Sample collection, isolation, DNA extraction, and genome sequencing

Strains of *Escovopsis* (six), *Luteomyces* (two), *Sympodiorosea* (three), and one undescribed genus (three) were obtained from the Emory collection (Table S1). To obtain DNA, fungi were grown on potato dextrose agar (PDA) plates at room temperature. Genomic DNA was extracted by crushing fungal tissue with liquid nitrogen and subsequently isolating the DNA using a phenol-chloroform protocol (34, 35). Sequencing was performed on a HiSeq 2500 Sequencing system from Illumina, utilizing the paired-end 150 bp technology. Both library preparation and DNA sequencing were carried out at Novogene. Additionally, DNA from strains NGL095 (*E. weberi*)*,* NGL070 (*Escovopsis multiformis*), and NGL057 (*Luteomyces* sp.) were also sequenced with PacBio Technology by Omega Bioservices.

### Genome assembly and annotation

Strains sequenced with PacBio Technology were assembled with Canu v.1.8 (36) and polished with their corresponding Illumina reads using Pilon v.1.23 (37). The strains sequenced with Illumina alone were quality checked with FastQC (38), trimmed with Trimmomatic (39), and subsequently assembled with Spades v.3.13.0 (40). Genome assembly quality was evaluated using Benchmarking Universal Single-Copy Orthologs (BUSCO) v.3 (41). GC content was calculated with the script GC_content.pl by Damien Richard [https://github.com/DamienFr/GC_content_in_sliding_window/ (last accessed July 2023)], using default parameters. The genomic data set was completed with the addition of 24 previously sequenced *Escovopsis* genomes (31, 33), as well as 14 closely related species from the *Hypocreales* obtained from JGI Mycocosm (Table S1). The highly contiguous hybrid assemblies NGL070, NGL095, and NGL057 were screened for stretches of telomeric repeats (TTAGGG)n at the end of contigs, and contigs harboring these repeats at both ends were considered complete chromosomes.

To compare genomic architecture conservation, a synteny analysis was performed on the proteome sets of the most unfragmented assemblies in our data set employing GENESPACE v.0.9.3 (42) as implemented in R. This data set comprised the three hybrid assemblies belonging to strains NGL095, NGL070 and NGL057, as well as EACOL, EAECHC, EAECHR, EPCORN, and EATTINE.

All assemblies were subjected to gene prediction and annotation using the Funannotate v.1.8.3 pipeline (43, 44). Repeats were identified with RepeatModeler and soft masked using RepeatMasker (45). Protein evidence from a UniprotKB/Swiss-Prot-curated database (46) and the proteomes from *Trichoderma* sp.*, Cladobotryum* sp.*, Hypomyces rosellus,* and *Hypomyces perniciosus* were aligned to the genomes using TBlastN and Exonerate (47). Three gene prediction tools were used: AUGUSTUS v.3.3.3 (48), snap (49), and GlimmerHMM v.3.0.4 (50). tRNAs were predicted with tRNAscan-SE (51). Consensus gene models were found with EvidenceModeler (52). Functional annotation was conducted using BlastP to search the UniprotKB/Swiss-Prot protein database. Protein families and Gene Ontology terms were assigned with InterProScan 5 (53). Additional predictions were inferred by alignments to the eggnog orthology database (54), using emapper v.3 (55). The secretome was predicted using Phobius v.1.01 (56), which identifies proteins carrying a signal peptide. Carbohydrate active enzymes were identified using HMMER v.3.3 (57) and family-specific HMM profiles of the dbCAN2 server (58). Proteases and protease inhibitors were predicted using the MEROPS database (59), and biosynthetic gene clusters were annotated using fungiSMASH v.6 (60) with relaxed parameters. Gene density was calculated for the highly contiguous strain NGL070 with the R package RIdeogram in R (61, 62), as the number of genes per 1 Mb window, and was visualized in an ideogram highlighting the number of genes per 100 Kb window, employing the same software.

### Phylogenetic reconstruction

Phylogenetic relationships were reconstructed using the BUSCO_phylogenomics pipeline (63). In short, single-copy orthologs for each genome were identified by running BUSCO v.5 (41) with the Ascomycota_odb10 lineage database. This analysis identified 660 single-copy orthologs shared by all 34 strains in the data set. Gene sequences were aligned with MUSCLE (64), and the alignment was trimmed with TrimAl (65). Output alignments were concatenated into a supermatrix. A maximum likelihood tree was built with IQ-TREE (66), allowing ModelFinder (67) to predict the best evolutionary model for partitioning the alignment. The resulting tree was rooted using *Trichoderma* spp. and visualized with iTol v.6 (68).

To place the genome-sequenced strains in a broader phylogenetic context, we performed a multi-locus analysis using three molecular markers: Internal Transcribed Spacer, Transcription Elongator Factor, and Large Subunit of the rRNA (ITS, TEF, and LSU, respectively). Sequences of each molecular marker were aligned in MAFFT v.7 (69) separately, and concatenated using Winclada v.1.00.08 (70). We reconstructed the final tree using Bayesian inference in MrBayes v.3.2.2 (71). Two separate runs, each consisting of three hot chains and one cold chain, were carried out using the GTR model (General Time-Reversible model) for each partition independently. The nucleotide substitution model was selected using jModelTest2 (72) with the Akaike information criterion and 95% confidence intervals. Five million generations of the Markov Chain Monte Carlo were necessary to reach convergence (standard deviation of split frequencies <0.01), and the first 25% of trees were discarded as burn-in to generate the best tree. *Lecanicillium antillanum* (CBS 35085) was used as the outgroup, and the final tree was edited in FigTree v.1.4.4 (http://tree.bio.ed.ac.uk/software/figtree/) and Adobe Illustrator 2023 v.28.0.

To estimate the evolutionary distance between strains, we performed a percentage of conserved proteins analysis (POCP) (73), using as input (i) the total number of proteins per species, and (ii) the Orthogroups_SpeciesOverlaps table obtained from an OrthoFinder (74) analysis, which contains the number of orthogroups shared between each species pair. The percentage of conserved proteins between two genomes was calculated with the following formula: [(C1 + C2) / (T1 + T2)] * 100, where C1 and C2 are the number of shared proteins in the two genomes being compared, respectively; and T1 and T2 are the total number of proteins in the two genomes being compared, respectively (73).

### Gene cluster family (GCF) identification

Biosynthetic gene clusters (BGCs) of all fungal strains were identified using fungiSMASH 6.1 (60) with relaxed parameters, utilizing as input the GenBank files obtained after genome annotation. With the aid of cblaster v.1.3.12 (75), BGCs split onto different contigs, especially those located on contig edges, were manually assembled based on homology with other BGCs in the data set. Likewise, fused BGCs were manually split into separate BGCs. The final BGC set was analyzed using BiG-SCAPE v.1.0.1 (76) to identify homologous BGCs across all strains and to cluster related BGCs into GCFs. BGCs from the MIBiG database 2.0 (77) were included in the analysis with the –mibig flag to identify already described BGCs. The scikit-learn package was downgraded to v.0.19.1, and the following parameters were enabled: –mix, --hybrids-off, and –include_singletons. The program was run in “glocal” alignment mode with edge-length cutoffs from 0.1 to 0.9, with step increments of 0.1. After inspection, networks at thresholds 0.5–0.6 were found to be similar and further analyses were based on a cutoff of 0.5. The resulting sequence similarity matrixes were visualized using Cytoscape v.3.9.0 (78). A presence/absence matrix was built to evaluate BGC distribution, with 1 representing presence and 0 representing absence of a GCF in a fungal strain and was visualized as a heatmap using R (62). To compare escovopsis’ BGCs to those already described and present in the MIBiG database, we employed cblaster (75). Using “cblaster makedb,” we created a local database consisting of GenBank files of all escovopsis BGCs. We subsequently employed “cblaster search” using the MIBiG clusters with homologous BGCs in our data set as queries to perform BLAST searches against the local database.

To assess whether BGC profiles can delineate groups of escovopsis symbionts, a Jaccard distance matrix was computed using the presence/absence table. The distance matrix was then used to construct nonmetric multidimensional scaling (NMDS) ordination plots to detect grouping patterns and subjected to an analysis of similarity (ANOSIM) and a permutational multivariate analysis of variance (PERMANOVA) to identify significant factors underlying observed groupings. To assess the adequacy of our sampling, and to provide an estimate of GCF richness for the given sequencing effort, rarefaction curves were built at the genus level, and at both levels of attine agricultural systems (i.e., lower and higher agriculture, as well as lower, coral, general higher, and leaf-cutter agriculture).

### Co-cladogenesis analyses

The GCF presence/absence was subjected to a hierarchical clustering analysis using a correlation-centered similarity metric with the complete linkage clustering method. A tanglegram was built in R (62) to evaluate the congruency between the symbiont phylogeny and strain BGC profiles using the package “dendextend” v.1.17.1.

### Ancestral state reconstruction

To assess the evolutionary history of the GCFs, the ancestral node for each GCF was inferred in the species tree using the trace character history function implemented in Mesquite (79). In some cases, BiG-SCAPE split BGCs into multiple GCFs that were highly similar, sharing many homologous genes, suggesting they may be involved in the biosynthesis of related compounds. Data exploration with different BiG-SCAPE similarity cutoffs did not resolve these relationships, prompting the manual grouping of GCFs into pathways (80, 81). GCFs were considered to belong to the same pathway if (i) the BGCs shared similar architecture (i.e., genes and other features arranged in similar ways), (ii) the majority of the genes in the cluster had the same function, albeit not necessarily in the same order, and (iii) the majority of genes in the BGC had a BLAST similarity of more than 50% over 80% coverage rate (81). A pathway presence/absence table was used as a character matrix, and likelihood calculations were performed using the Mk1 model. Likelihood scores >50% were used to infer the points of pathway acquisition in the species tree.

### Statistical analyses

All statistical analyses were carried out in R. v.4.1.1 (62). Differences in genome size across escovopsis strains were analyzed using a general linear model after data transformation and validation of a normal distribution, as well as a phylogenetic ANOVA. A non-parametric Wilcoxon rank sum test and phylogenetic ANOVAs were employed to assess differences in gene, transposable elements, and BGC content. The correlation between gene content and genome size was analyzed employing a phylogenetic generalized least square model (PGLS) in which we assumed a strict Brownian model of gradual evolution for strains, with branch lengths being proportional to the amount of evolutionary change (82). The phylogenomic tree employed for this analysis was built using a proteome data set comprised of single-copy genes of 112 fungal strains [including all strains in our analysis and other *Sordariomycetes* (Table S2)]. Protein sequences were aligned with MUSCLE (64), and the alignment was trimmed with TrimAl (65). Output alignments were concatenated into a supermatrix. The phylogeny was built with FastTree (83). Further statistical details for each test can be found in the main text and in Table S3. For every statistical analysis, significance was defined as *P* ≤ 0.05.

## RESULTS AND DISCUSSION

To characterize the genomic features and secondary metabolism potential of this diverse group of specialized symbionts*,* we sequenced the genomes of 14 strains across the symbiont phylogeny, spanning all ant agriculture ecologies (Table S1) (25), with the exception of yeast agriculture, where escovopsis symbionts have never been found. Three strains (NGL057, NGL070, and NGL095) belonging to different clades were sequenced with PacBio and Illumina Technologies, whereas the rest were sequenced with Illumina alone (Table S1). We expanded our data set with the addition of genomes of 24 strains previously classified as *Escovopsis*, that were publicly available (31, 33), and genomes of a number of other closely related fungal species from the *Hypocreaceae* (Table S1).

The quality of the genomic assemblies generated in this study was high, with an average BUSCO score of 94.7% for the *Ascomycota* lineage data set (Table S1). GC content ranged from 47.2% to 56.4%, with an average of 52.3% (Table S1), consistent with recent reports (29, 33) and other *Pezizomycotina* fungi (84).

### Phylogenetics of *Escovopsis* and relatives

**Fig 1:**
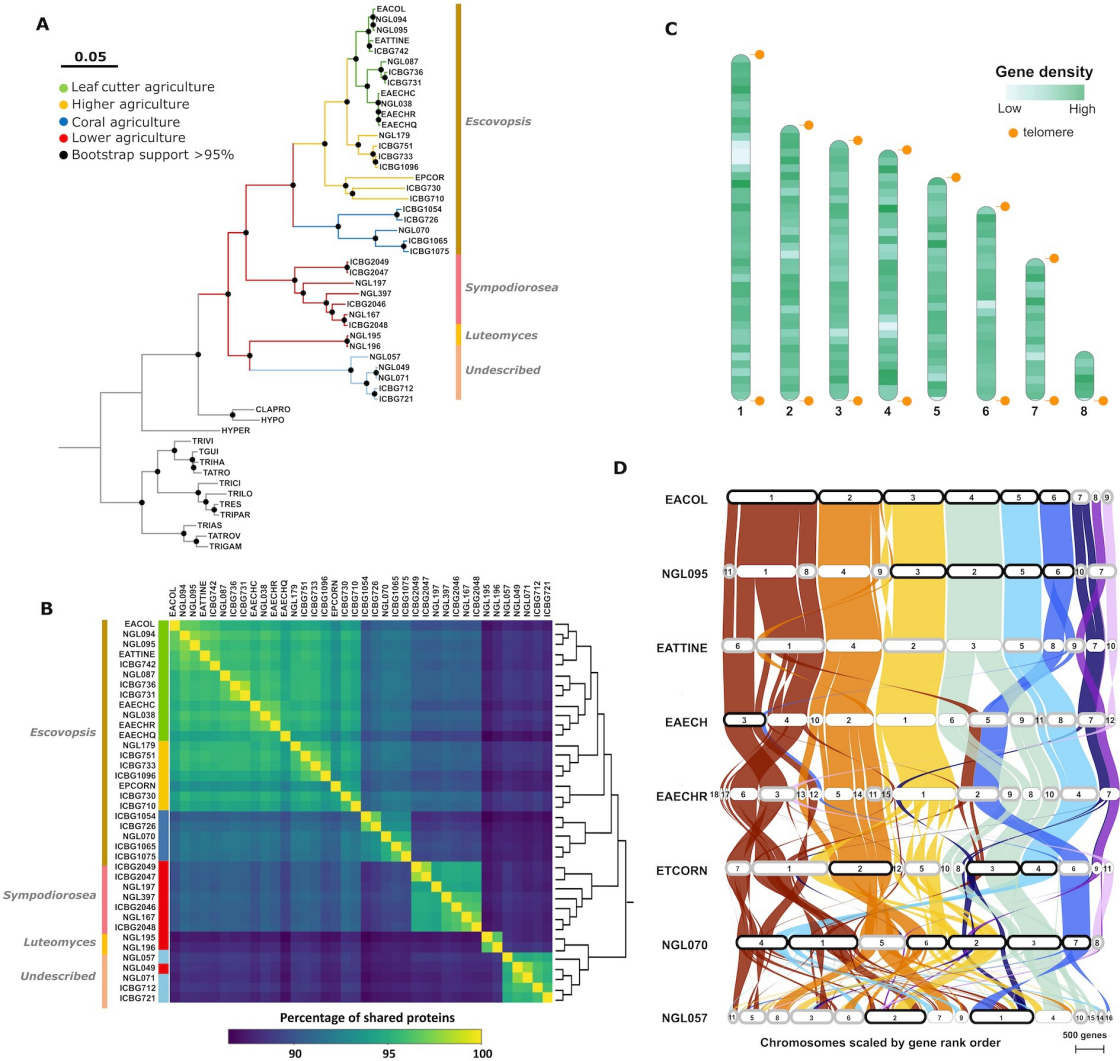
Genomic features of *Escovopsis* and allies. (**A**) Phylogenomic tree constructed with a supermatrix approach on 2,314 single-copy orthologous genes. Black dots represent bootstrap support higher than 90%. Branch colors describe different attine agricultural systems: green, leaf-cutter agriculture; yellow, general higher agriculture; blue, coral agriculture (divided into dark blue and light blue to represent distinct clades, respectively); and red, lower agriculture. Side colored bars represent taxonomical affiliations based on reference (14). (**B**) Heatmap depicting the percentage of conserved proteins across strains. Lighter colors represent high levels of shared proteins, whereas dark colors depict fewer shared proteins. The dendrogram on the right represents a hierarchical clustering analysis. (**C**) Ideogram representing the chromosomal level assembly of an *Escovopsis* sp. strain isolated from an *Apterostigma dentigerum* nest (NGL070). Light and dark green colored bands represent regions with low and high gene density (ranging from 1 to 43 genes per 100 Kb window). Orange dots represent areas harboring telomeric repeats. (**D**) Synteny plot depicting the collinearity between the seven most continuous *Escovopsis* genomes (EACOL to NGL070) and one *Luteomyces* genome (NGL057) available across attine agriculture. Highly syntenic regions are connected by colored bands. Contigs in black boxes represent complete chromosomes, whereas those in gray harbor telomeric repeats just at one chromosomal end.

To infer a genome-scale phylogeny of representative *Escovopsis, Sympodiorosea, Luteomyces,* and relatives, we employed a concatenation approach using single-copy genes. The inferred proteomes of all 52 species in our data set were subjected to an orthology analysis, resulting in 2,314 single-copy orthologous genes that were subsequently utilized to infer a phylogeny. The resulting phylogeny reveals that the attine-associated symbionts form a monophyletic group, sister to a clade composed of *Cladobotryum* sp. and *Hypomyces rosellus*, both mycoparasites (Fig. 1A). The evolutionary history of the attine-associated symbionts suggested by this phylogeny generally reflects that of the ants (25). As such, strains infecting gardens of lower attines appear as a sister group to the rest, whereas most recently diverging lineages are associated with higher attine agriculture and leaf-cutter ants (Fig. 1A). The shift experienced by some lower attines to cultivating *Pterulaceae* fungi is also mirrored by the phylogeny, with an intermediate clade exploiting coral agriculture, represented by strains NGL070, ICBG726, ICBG1054, ICBG1065, and ICBG1075. Highlighting the diversity of symbionts associated with coral agriculture, a clade including four strains associated with coral fungi (ICBG712, ICBG721, NGL057, and NGL216) appears within the basal members of this monophyletic group (Fig. 1A). The presence of these two distinct coral agriculture-associated clades, therefore, break congruence of the ant and symbiont phylogenies. Recent studies have split the genus *Escovopsis* intro three different genera (*Escovopsis*, *Sympodiorosea*, and *Luteomyces*) based on key morphological differences and phylogenetics using five fungal molecular markers (24). To assess whether these two coral agriculture-associated clades may in fact represent two putative distinct taxonomical genera, we inferred the phylogenetic position of the escovopsis symbionts in this study among those from previous studies (24). Our results (Fig. S1) suggest that strains within these two clades indeed belong to different genera. Together with strains exploiting higher agriculture (*E. weberi, Escovopsis moelleri*, and *Escovopsis aspergilloides*), the intermediate clade exploiting coral agriculture are true *Escovopsis* (*E. multiformis*). However, its sister clade contains strains closely related to the newly described *Sympodiorosea*. Interestingly, the sister clade to that containing *Escovopsis* and *Sympodiorosea* comprises strains most closely related to *Luteomyces* and to strains belonging to a yet undescribed genus (Fig. S1). Overall, these results highlight the need for further work to fully resolve the taxonomical diversity within this symbiont group.

To estimate the evolutionary distance between strains, we performed a POCP analysis (73). As expected, with increased phylogenetic distance, POCP values decrease. For instance, *Escovopsis* spp. infecting leaf-cutter agriculture share, on average, 96% of their proteins among each other, whereas only around 88% are shared with *Luteomyces* spp., *Sympodiorosea* spp., and strains within the newly undescribed genus (Table S4; Fig. 1B). Despite appearing in the same clade in our phylogeny, *Luteomyces* and the undescribed genus share as many proteins between each other (88%) as each of these genera do with strains infecting any other agricultural system. This suggests that there is as much phylogenetic divergence between these two groups as there is between them and any other clade, supporting the notion that what has been traditionally considered *Escovopsis* is in fact at least three, and possibly four, different genera. Furthermore, POCP values lower than 91% segregate our data set into the recently proposed genera, whereas values above 91% and 95% delineate distinct species and strains within a species, respectively (Table S4). Mirroring our phylogenetic placement of *Mycetomoellerius zeteki*-associated *Escovopsis*, in POCP analysis, NGL179 shares more proteins (95.1%) with strains infecting leaf-cutter agriculture than with those exploiting general higher agriculture (92%). POCP analyses have been useful to resolve bacterial groups at genus level, which correlate with POCP values <50%. While some studies have implemented the method in fungi at the family level (POCP values <70%) (70), this strategy cannot be widely employed yet for delineating fungal groups, as genome sampling in fungi remains scarce. However, our POCP analysis reveals a significant degree of genetic diversity between escovopsis clades and suggests a protein similarity threshold of 87%–91% to delineate different genera in this group of symbionts. Further efforts are required to elucidate whether the POCP differences can delineate distinct genera in a diversity of fungi.

### Genomes are organized into highly syntenic chromosomes

To elucidate the genomic organization of these symbionts, we screened the genomes of the four most contiguous assemblies for telomeric repeats. In *Escovopsis* sp. NGL070, stretches of (TTAGGG)n were detected at both ends of six contigs, representing complete chromosomes (Fig. 1C). The two remaining contigs harbored telomeric repeats only at one end, constituting either two fragments of the same chromosome, or two distinct incomplete chromosomes. A similar pattern was observed for the highly contiguous *Escovopsis* sp. EACOL, *Escovopsis* sp. NGL095, and *Luteomyces* sp. NGL057 genomes assemblies, harboring six, four, and two complete chromosomes and two, five, and seven fragmented ones with telomeric repeats at one end, respectively (Fig. 1D). These observations suggest that these symbionts have seven to eight chromosomes, in agreement with other members of the *Hypocreales* order, such as *Trichoderma reesei, Neurospora crassa* (85), and *Metarhizium brunneum* (86), which organize their genomes in seven chromosomes.

To assess the conservation of genomic architecture across this diverse group of symbionts, we performed a synteny analysis of the eight most continuous genomes available. Our ortholog-based analysis reveals that strains share a high degree of collinearity, with 87.83% of the genes appearing in the same chromosome and in the same order (Fig. 1D). This is particularly apparent among strains of the same clade, as evidenced by *Escovopsis* spp. associated with leaf-cutter agriculture (EACOL, NGL095, EATTINE, EAECHC, and EAECHR). As expected, collinearity has a positive correlation with phylogenetic relatedness, with distant strains exhibiting increasingly different genomic organization. Chromosomes 1, 2, 3, 4, and 5 (nomenclature relative to strain EACOL) are extremely well conserved, extending beyond *Escovopsis* spp. infecting leaf-agriculture and including those involved in general higher agriculture. Chromosome 6, although well conserved in *Escovopsis* spp. affiliated with general higher agriculture and coral agriculture-associated NGL070, has experienced recent rearrangements, as evidenced by its fusion with a fragment of chromosome 1 occurring in the clade represented by EAECHC and EAECHR. Previous reports revealed a high degree of microsynteny and mesosynteny between genomes of *Escovopsis* and *Trichoderma* (29), suggesting that both genomes are organized in genome segments with similar gene content but rearranged in order and orientation.

### Symbionts have reduced genomes

**Fig 2:**
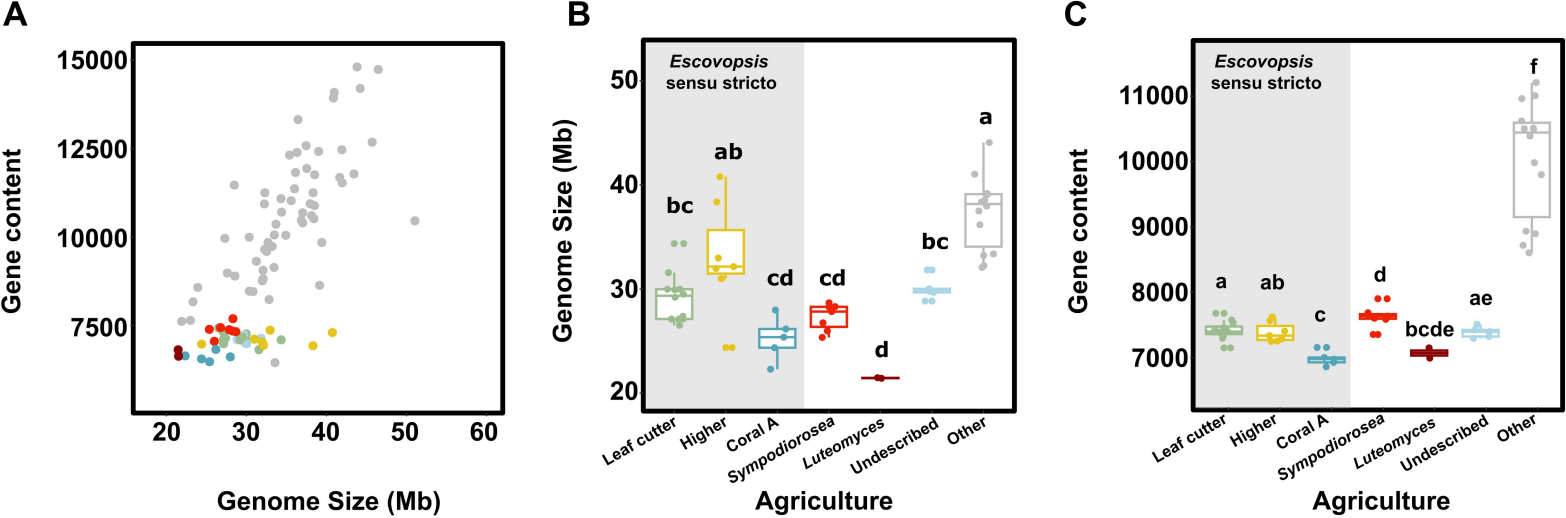
*Escovopsis* and related symbionts harbor reduced genomes with fewer genes than their non-ant associated relatives. (**A**) Relationship between genome size and gene content for sequenced fungal genomes. Genome size (**B**) and gene content (**C**) of escovopsis symbiont strains across different attine agricultural systems. Box colors denote attine clades systems: green, leaf-cutter agriculture (*Escovopsis* spp.); yellow, general higher agriculture (*Escovopsis* spp.); blue, coral agriculture A (*Escovopsis* spp.); red, lower agriculture (*Sympodiorosea* spp.); dark red, lower agriculture (*Luteomyces* spp.); light blue, coral agriculture B (undescribed genus); gray, other *Sordariomycetes*.

Fungi vary extensively in genome size, spanning three orders of magnitude and ranging from the small genomes of some Microsporidia (2 Mb) to the large ones in Pucciniales fungi (2 Gb). Some of the smallest genomes are found in obligate parasites (87). Escovopsis symbiont genome sizes range between 21.4 Mb and 38.3 Mb (40.7 Mb), with an average of 28.7 Mb, corroborating previous studies (29, 33) that estimated their genome sizes around 24.7 Mb–27.2 Mb. These genomes are reduced in size relative to those of closely related *Sordariomycetes* (Fig. 2A and B; Fig. S2A and B and Table S3). Interestingly, escovopsis symbionts represent three of the five smallest genomes from all *Sordariomycetes* strains publicly available in Mycocosm (https://mycocosm.jgi.doe.gov) (Fig. 2A). The other two belong to *Ophiocordyceps camponoti-rufipedis* and *Ophiocordyceps australis* strain 1348a, both highly specific parasites of ants (88). Within the attine-associated symbionts, lower attine *Luteomyces* spp. strains harbor significantly smaller genomes than those infecting higher attine nests (Fig. 2B; Table S3). No differences in genome size were detected across the other clades (Fig. 2B; Table S3), though, notably, *Escovopsis* spp. infecting higher agriculture vary greatly in genome size.

Gene number in escovopsis symbionts ranged between 6477 and 7693 (Table S1), representing 9 out of the 10 species in Mycocosm with the fewest genes within the *Sordariomycetes* (Table S2). Unlike other fungi in the family, where gene content positively correlates with genome size (*r*^2^ = 0.32, *P* < 0.0001; PGLS, *P* < 0.0001, Table S3), gene number in escovopsis symbionts is stable and does not associate with genome size (*r*^2^ = 0.06, *P* = 0.07; PGLS *P* = 0.21, Table S3) (Fig. 2A). While escovopsis symbionts harbor fewer genes than their relatives (Kruskal-Wallis rank sum test, χ^2^ = 30.11, d.f. = 1, *P* < 0.001; phyloANOVA *P* < 0.001, Fig. S3A; Table S3), there is no difference in gene content between symbionts exploiting the nests of lower and higher attines (Table S3; Fig. S3B). However, those *Escovopsis* spp. associated with coral agriculture (“Coral clade A”) have a slightly lower gene content than other *Escovopsis* spp., *Sympodiorosea* spp., and species of an undescribed genus (Fig. 2C; Table S3). These results are congruent with a recent survey (33) revealing that total coding sequences length and intron number in escovopsis symbiont genomes are low in comparison to free-living relatives, consistent with reduced gene content. Escovopsis symbionts present an average gene density of 292 genes per Mb, only slightly higher than that of other ascomycete fungal symbionts of insects such as *Metarhizium acridum* (259), *Metarhizium anisopliae* (271), *Cordyceps militaris* (257), and the palm aphid YLS (274) (89).

In addition to gene number, we investigated two drivers of fungal genome size: repeat content and repeat-induced point mutation (RIP). First, while transposable elements are often associated with fungal pathogens (90, 91), their number in escovopsis symbiont genomes is significantly lower than in non-ant-associated relatives (Kruskal-Wallis, ^χ2^ = 14.19, d.f. = 1, *P* < 0.001), which in part explains the symbionts’ small genomes. Second, fungi have evolved a genome defense mechanism to mitigate the potentially detrimental consequences of transposable elements and other repeated genomic regions (92). By altering nucleotide ratios, RIP can inactivate duplicated genes that can be subsequently purged through selection, potentially contributing to genome reduction. Deactivation of RIP, therefore, can lead to genome expansion due to retrotransposon proliferation (93). Previous reports based on the analysis of a single strain of *E. weberi* suggested that it may have lost genes involved in RIP (29). BLAST analyses with the sequences of the two canonical genes known to mediate the RIP pathway (94, 95) revealed that all attine-associated symbiont genomes in our data set harbored orthologs for one gene essential to the RIP process (RID, RIP deficient) but lacked orthologs to the other RIP canonical gene (DIM2, defective in methylation) (Tables S5 and S6). Genome-wide RIP analyses using the RIPper’s sliding window approach revealed that all escovopsis symbiont strains show hallmarks of RIP (Table S7), although they vary greatly in the proportion of their genomes that are affected by it. While some strains harbored little evidence of RIP (ICBG1096, 1.01%), others are highly affected by it, with the most extreme case being ICBG1075, where 23.26% of its genome present hallmarks of RIP. This variation across genomes of similar size indicates that RIP is not solely responsible for genome reduction in this group of symbionts, but it may play some role in some species. While RIP processes require sexual recombination (96), most escovopsis symbiont genomes lack complete fungal mating-type loci (Table S8), suggesting they cannot undergo sexual reproduction and may therefore be uncapable of carrying out RIP.

These symbionts’ small genomes and the genomic traces of RIP, together with the presence of RID, support previous studies (29) that proposed RIP as a genomic defensive mechanism that limited transposon proliferation in *Escovopsis* spp. in the past. A consequence of RIP is the relative absence of duplicated genes (92). Therefore, the loss of this defense mechanism may represent an opportunity for these symbionts to evolve new metabolic functions through gene duplication and subfunctionalization.

Ascomycota with genome sizes between 25 and 70 Mb, and in particular *Sordariomycetes*, often exhibit positive correlations between genome size and gene content (87, 97, 98). These attine-associated symbionts evade this trend (Fig. 2A), suggesting that different evolutionary processes may be affecting this group. Symbiosis often leads to the streamlining of microbial genomes through genome reduction and gene loss, as epitomized by the tiny genomes of many bacterial endosymbionts of insects (99). Genome streamlining in bacteria can be explained by the loss of redundant genes with drift (100), or by selection against non-essential genes (101). Similar dynamics can occur in fungal mutualists and parasites (102). In particular, fungal parasites associated with insects have been shown to be particularly prone to gene loss (98). Within the *Sordariomycetes*, the smallest genomes belong almost exclusively to endosymbionts, endoparasites, or fungal parasites vectored by insects (98). In other eukaryotic microbes such as *Microsporidia* obligate parasites, genome reduction and gene loss correlate with accelerated rates of molecular evolution (103, 104). It is still unknown whether similar processes are occurring in escovopsis symbionts, as suggested by longer branches in the phylogenetic tree relative to those in their close relatives (Fig. 1A).

### BGC diversity and distribution

Secondary metabolites in fungi can define ecological niches (105), delimit host ranges (4, 106, 107), and provide selective advantages under specific ecological conditions (108). The metabolic pathways responsible for the synthesis of microbial toxins and other secondary metabolites are typically encoded by BGCs. BGCs encode for backbone enzymes responsible for the synthesis of the core structure of a metabolite, as well as tailoring enzymes that modify this assembly, along with transcription factors and transporters (109). To assess the biosynthetic potential of these symbionts, we performed a computational genome mining analysis using the program fungiSMASH (60). The most common backbone enzymes in fungi include polyketide synthases (PKSs), nonribosomal peptide synthetases (NRPSs), terpene synthases, and dimethylallyltransferases (110). All genomes analyzed harbored a diversity of BGCs belonging to the major biosynthetic classes (Table S9). The symbionts’ chemical potential contents ranged from 16 BGCs in *Sympodiorosea* sp. NGL197, to 33 in *Luteomyces* sp. NGL057. On average, each genome featured 23 BGCs, and an average metabolic diversity of 28.7% NRPs, 25.6% PKS, 21.3% terpenoids, 16.3% hybrids, 2.4% betalactones, and 3.6% others. There was no correlation between the number of BGCs and the number of contigs or scaffolds per genome (*R*^2^ = 0.03, *P* = 0.13), suggesting that our data set was robust and that the different sequencing technologies employed did not bias our BGC survey. In addition, no correlation was found between the number of BGCs in each strain and genome size (*R*^2^ = 0.004, *P* = 0.7).

**Fig 3:**
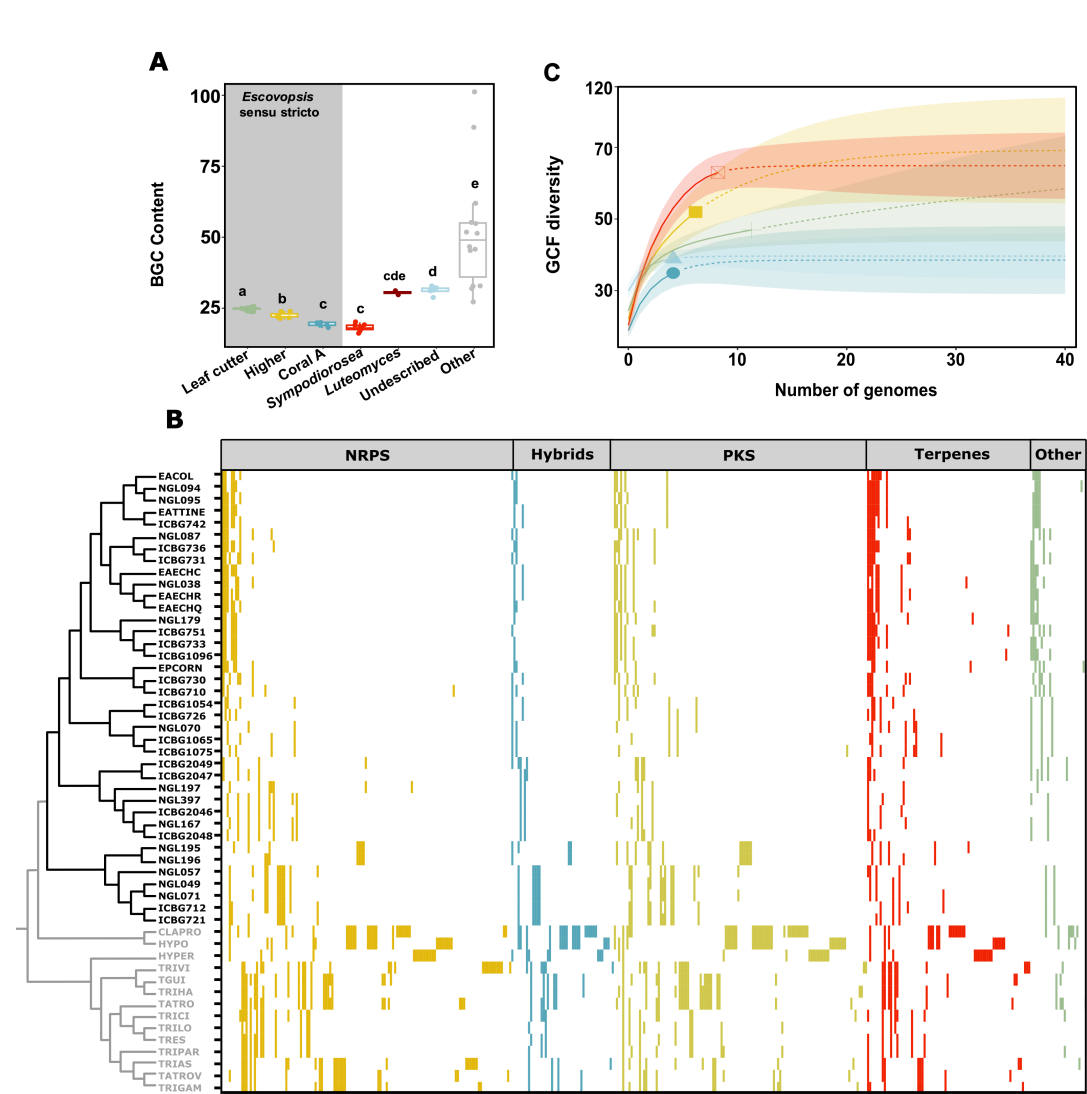
(**A**) Total number of BGCs identified across symbiont strains infecting different attine agricultural systems and non-ant-associated relatives. Box colors denote attine clades systems: gray, free-living; green, leaf-cutter agriculture (*Escovopsis* spp.); yellow, general higher agriculture (*Escovopsis* spp.); blue, coral agriculture A (*Escovopsis* spp.); red, lower agriculture (*Sympodiorosea* spp.); dark red, lower agriculture (*Luteomyces* spp.); and light blue, coral agriculture B (undescribed genus). (**B**) GCF distribution across symbionts. Each column in the heatmap represents a GCF. The presence of a GCF in a strain is highlighted by colored blocks according to BGC class: yellow, NRPs blue, PKS-NRPS hybrids; light green, PKS; red, terpenes; and green, others (including RiPPs, indoles, siderophores, and others). The absence of a GCF is represented by white spaces. (**C**) Rarefaction curves assessing GCF richness in symbiont strains across different attine agricultural systems for the given sequencing effort. Continuous lines represent observed diversity, dashed lines inferred diversity. Shaded areas denote confidence intervals. Colors denote agricultural systems: green, leaf-cutter agriculture; yellow, general higher agriculture; blue, coral agriculture A; red, lower agriculture; and light blue, coral agriculture B.

While fungi within the *Hypocreales* are prolific secondary metabolite producers, with an average of 43 BGCs per genome, escovopsis symbionts have significantly fewer BGCs than their non-fungus-farming ant-associated relatives (Kruskal-Wallis χ^2^ = 28.17, d.f. = 1, *P* < 0.001, Fig. S4A), corroborating recent findings using fewer escovopsis symbiont genomes (33). We found no statistical differences in BGC abundance between strains infecting higher or lower attine nests (Fig. S4B), nor between the majority of strains associated with different agricultural systems (Kruskal-Wallis *P* = 0.67, Fig. 3A), with the exception of small differences in BGC number in strains infecting general higher agriculture and leaf-cutter agriculture. Upon graphical inspection, we observed a clear bimodal distribution in BGC abundance in strains infecting lower agriculture (Fig. 3A) that unequivocally divided the data set into distinct phylogenetic taxa. We therefore explored whether there is a correlation between BGC content and phylogeny by assessing differences in BGC number across clades (Fig. S4C). All clades harbored significantly different number of BGCs, with the exception of *Luteomyces* spp. and the undescribed genus, both composed of strains infecting lower agriculture, which were similar to each other (Kruskal-Wallis, χ^2^ = 47.91, d.f. = 6, *P* < 0.001). Strains within *Luteomyces* and the undescribed genus (i.e., NGL195, NGL196, NGL049, NGL057, NGL216, ICBG712, and ICBG721) harbor more BGCs than more derived strains. Within *Escovopsis* spp., there is an increase in BGC abundance from those strains associated with lower agriculture (coral A) to those associated with the more derived (leaf-cutter agriculture) (Fig. S4C). As escovopsis groups within our data set strongly correlate with genetic distance, phylogenetic ANOVAs are not significant (Table S3, phyloANOVA *P* > 0.05). These patterns suggest that relatedness shapes differences in BGC content.

The reduction in BGC abundance in these symbionts relative to other non-ant-associated *Hypocreales* is consistent with a shift in lifestyle to being obligate symbionts of ant gardens. Transitions from free-living states to obligate symbioses can often be accompanied by gene loss due to relaxed selection on genes that are no longer necessary in a stable, predictable environment (29, 111). Additionally, some specialist parasites are known to harbor a narrower suite of BGCs relative to generalist ones. For instance, *Metarhizium* strains that acquired the *dtx* biosynthetic gene cluster, responsible for the synthesis of a diversity of toxins, have broader host ranges (infecting hundreds of insect species) compared with non-toxigenic strains (lacking the BGC), which have much narrower host ranges, infecting only locusts and grasshoppers (4). Correlating with a higher content of biosynthetic gene clusters, *Escovopsis* spp. strains infecting higher agriculture (e.g., *E. weberi*) are thought to be more virulent than the symbionts infecting lower agriculture (22).

To compare BGC composition across all strains (including all escovopsis symbiont strains and other *Hyypocreaceae* strains), we grouped BGCs into GCFs based on sequence homology and cluster architecture employing the BiG-SCAPE algorithm. The resulting sequence similarity network built with a similarity score cutoff of 0.5, clustered 1,595 BGCs into 415 GCFs. We visualized the GCF distribution across the symbionts through the construction of a presence/absence table (Fig. 3B). One hundred twenty-eight GCFs were present in the sampled escovopsis symbionts, and 102 of them were unique to the attine-associated symbionts relative to non-attine-associated fungi. Only 26 GCFs were shared between the symbionts and other *Hypocreales* species (Table S10; Fig. 3B). A rank-abundance curve demonstrates that 27 GCFs occur only once in the escovopsis symbionts, and an additional 27 are present in just two strains (Fig. S5). Surprisingly, no GCF as defined by Big-SCAPE was ubiquitous across all the symbiont strains*,* and therefore characteristic of the group of symbionts as a whole. Rarefaction curves provide an assessment of GCFs richness for the given sequencing effort and reveal that although our sampling was largely adequate, additional chemical diversity is yet to be discovered, especially within the undescribed genus infecting lower attine gardens (Fig. 3C). Further sequencing efforts in strains from this group may reveal additional GCFs.

To distinguish novel BGCs from already described ones, we supplemented our data set with characterized gene clusters from the MIBiG database as a reference, which at the date of analysis contained 1,923 BGCs, out of which 207 were of fungal origin. Given that recent surveys reveal that less than 3% of the biosynthetic space represented by fungal genomes has been linked to metabolites (110, 112), it is not surprising that only five GCFs in our symbiont data set are homologous to BGCs in the database. Three families comprising highly similar BGCs group together with the MIBiG cluster BGC0001585, responsible for the synthesis of melinacidin IV, suggesting they represent slightly different variants of the same biosynthetic pathway. The other two GCFs are homologous to BGC0001583 and BGC0001777, which potentially encode for emodin and shearinines, respectively. The similarity between escovopsis symbionts’ genes within BGCs and their homologs in the MIBiG database range between 50.1% and 100%, with an average of 87% (Table S11). Likewise, the majority of the symbionts’ BGCs harbors all the genes present in the MIBiG BGCs (Table S11). The distribution of all three GCFs is discrete. While most attine-associated symbiont strains harbor the BGC responsible for the production of melinacidin IV, those encoding for shearinine and emodin are restricted to more derived clades (i.e., *Escovopsis* spp. for shearinine, and *Escovopsis* spp, with the exception of those exploiting coral agriculture, for emodin).

Fermentation experiments using *E. weberi* have led to the detection and elucidation of the potential functional role of all three metabolites and some derivatives (31). *E. weberi*-produced shearinine derivatives can deter ants and are lethal at high concentrations, preventing insect workers from weeding their garden, thus allowing the parasite to persist in the nest (31). The production of epipolythiodiketopiperazine melinacidin IV inhibits the growth of the ant-defensive mutualist *Pseudonocardia*, whereas the synthesis of emodin has detrimental effects on the cultivar (31) and other co-occurring Actinobacteria, such as *Streptomyces*. While the production of these metabolites has been detected in *Escovopsis* strains parasitizing leaf-cutter ant gardens, our results demonstrate that the distribution of these BGCs is broader than previously thought and extends to strains exploiting other agricultural systems. Whereas shared GCFs with other fungal genera suggest that they may play a general role in fungal physiology, the presence of GCFs characteristic of specific clades correlating with different attine agricultural systems likely reflects the distinct selective pressures exerted on the symbionts by these different ecosystems. These results are consistent with an ongoing arms-race in which these symbionts must constantly evolve new adaptations to overcome not only cultivar defenses but also, very likely, ant defenses, those exerted by protective symbionts such as *Pseudonocardia* and those exerted by other microbes that inhabit these complex microbial communities. For example, the defensive symbiont of beewolves, *Streptomyces* spp., produces different antibiotic cocktails (both in composition and concentration) in association with each insect species, but also in distinct geographical regions (113), presumably as an adaptation to defend their hosts against different local pathogen communities. Furthermore, the varied metabolic profiles of these symbionts could be a reflection of them having different impacts on the agriculture system; while some (e.g., *E. weberi*) have been shown to be highly virulent parasites of the ants’ cultivars, experimental tests of the impacts of other species suggest low to no virulence (13–15, 21–23, 114, 115). More experimental work is required to assess the specific roles that individual metabolites may play in the ecology of this diverse group of symbionts.

### GCFs delineate groups of symbionts

**Fig 4:**
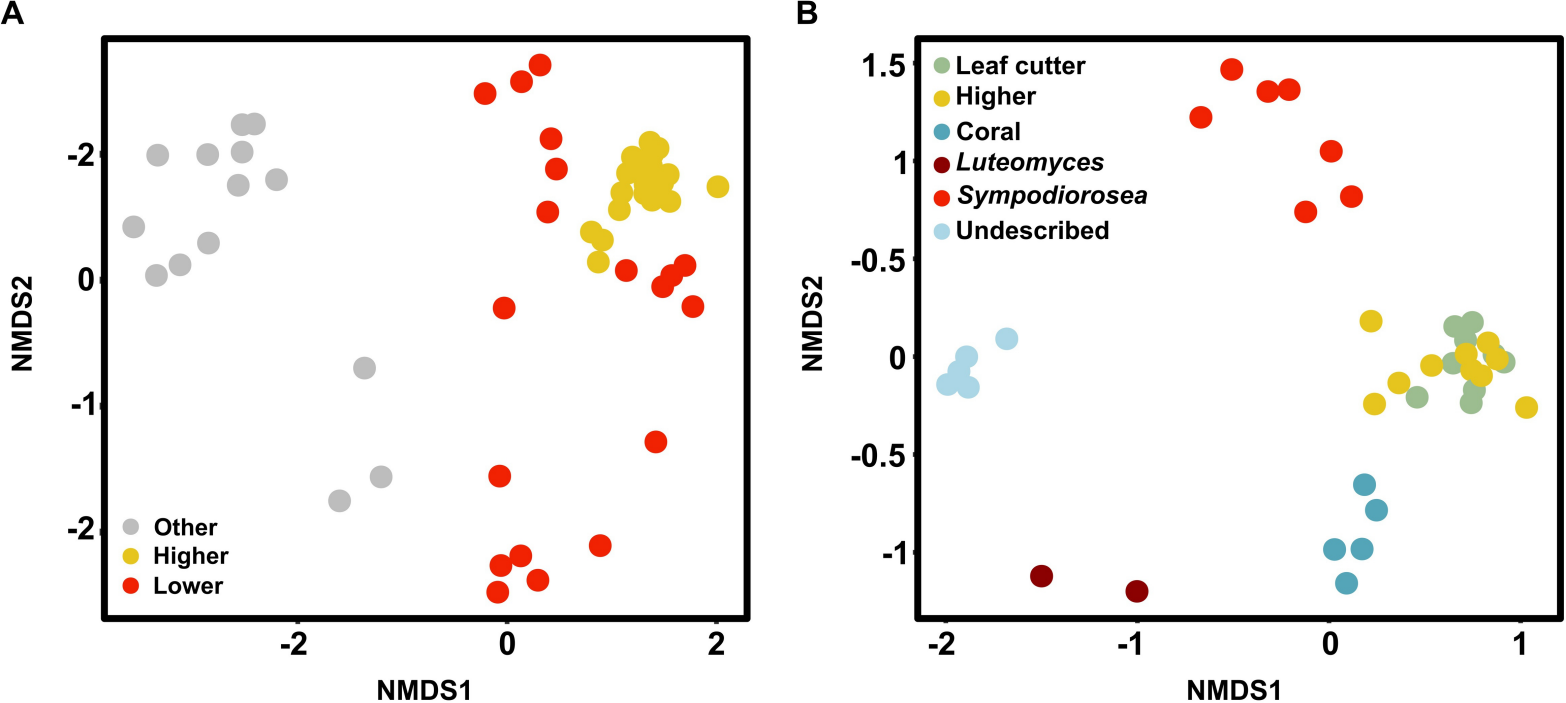
GCFs delineate symbiont groups. (**A**) NMDS plot showing differences in GCF composition among symbionts infecting higher agriculture (yellow) and lower agriculture (red) and non-ant-associated fungal relatives (gray). (**B**) NMDS plot depicting GCF composition of symbiont strains across ant clades: *Escovopsis* spp. (green, leaf-cutter agriculture; yellow, general higher agriculture; blue, coral agriculture A), dark red, *Luteomyces*; red, *Sympodiorosea*; and light blue, the undescribed genus.

To assess differences in biosynthetic profiles between symbiont strains associated with different attine agricultural systems, we performed a non-metric multidimensional scaling analysis. Our results demonstrate that the attine-associated symbionts harbor very different GCF profiles than related non-ant-associated fungi, and that these profiles differ between symbionts infecting higher and lower agriculture (Fig. 4A, ANOSIM, *R* = 0.68, *P* < 0.001, 999 permutations). Likewise, GCF profiles are sufficient to cluster strains into separate groups based on phylogenetic lineage (Fig. 4B, ANOSIM, *R* = 0.81, *P* < 0.001, 999 permutations). A PERMANOVA reveals that most of the variation (95%) is explained by the interaction between symbiont genus and ant species (Fig. 4B, adonis2, 999 permutations, *R*^2^ = 0.952, *P* = 0.001).

**Fig 5:**
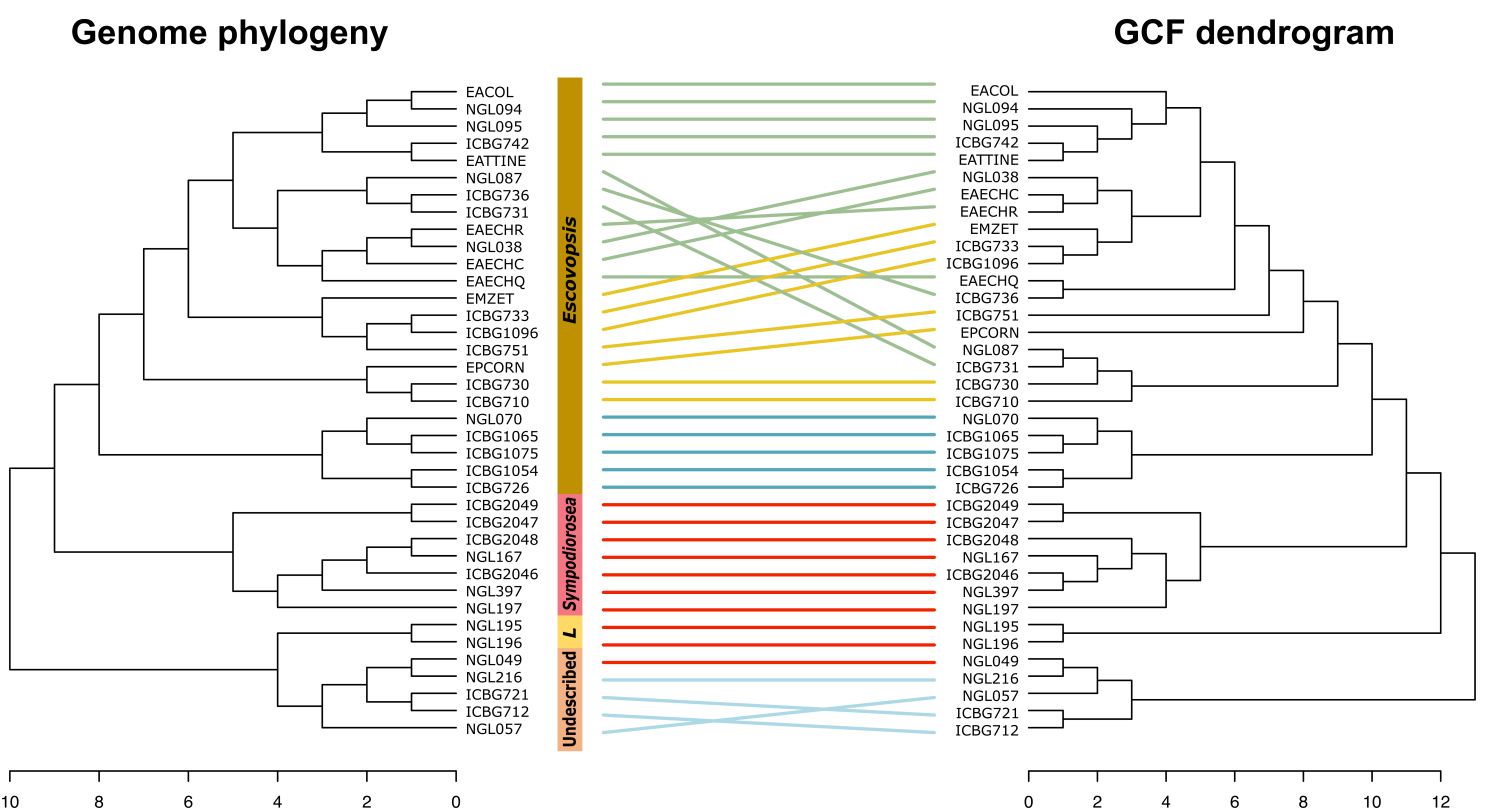
GCF profiles are a phylogenetic trait. Tanglegrams revealing congruence between symbiont genome phylogeny and biosynthetic potential. Lines connect strains with their GCF profile, and colors denote attine clades systems: green, leaf-cutter agriculture; yellow, general higher agriculture; blue, coral agriculture A; red, lower agriculture; and light blue, coral agriculture B. Branches have been rotated for maximum congruency. The maximum likelihood tree was built with 681 single-copy orthologous genes. The chemical dissimilarity dendrogram was generated using hierarchical cluster analysis on the presence and absence of GCFs using Jaccard distance and unweighted pair group method with arithmetic mean as the clustering method.

Based on the presence/absence matrix of GCFs across strains, we constructed a hierarchical clustering analysis. The symbiont genome phylogeny, based on all orthologs, and the GCF dendrogram are highly congruent (Fig. 5), with the exception that the clade comprising strains associated with coral agriculture and lower agriculture are paraphyletic in the GCF dendrogram. An entanglement analysis gives a visual approximation of the level of agreement between two dendrograms (116). A score of zero means no entanglement, or congruence, while one means full entanglement, or no congruence. Our analysis yielded a score of 0.02, suggesting a high degree of congruence between the symbiont genome phylogeny and the GCF dendrogram.

These results suggest that the symbionts’ biosynthetic potentials are phylogenetic traits and can be employed to delineate groups, particularly at broad taxonomical levels. Christopher et al. (115) demonstrated that phylogenetic analyses based on chemical profiles of escovopsis symbionts resulted in similar tree topologies to gene-based phylogenies, confirming that chemical profiles can be considered phylogenetic traits. Additionally, the congruency between the species phylogeny (Fig. S1) and the BGC profile dendrogram suggests that BGCs are evolving in parallel with the symbiont species, and that pathway gains and subsequent vertical inheritance, as well as losses, are the main forces driving BGC diversification, given that horizontal transfer of BGCs between escovopsis symbionts or with other fungi would result in incongruent topologies.

To further explore the possibility of vertical inheritance of BGCs in escovopsis symbionts, we evaluated whether strains missing a particular BGC still harbored orthologs to most genes in that BGC for a subset of five representative GCFs (Tables S12 through S16; Data sets S1 through S5). Our results suggest BGCs in escovopsis evolve vertically from standing genetic variation, given that lineage-specific GCFs such as BGCs potentially encoding for shearinine or emodin harbor genes with orthologs in species without that BGC (Tables S12 and S13, and Data sets 1 and 2, respectively). Often, the orthologs in BGC-lacking strains occur more dispersed in the genome relative to those in the BGC harboring strains and are not flanked by similar genes, suggesting that genomic rearrangements may have facilitated the evolution of such BGCs (Tables S12 through S16). Likewise, our analysis of single-gene phylogenies revealed that while genes within a BGC often evolved from ancestral gene duplications already present in the last common ancestor of all the strains present, they can also sometimes be a lineage-specific innovation. For example, while the majority of strains in our data set contain orthologs to most genes in the gene cluster family FAM_02655 (Table S10), the complete BGC only evolved after the lineage-specific acquisition of three genes, including the backbone gene, a terpene synthase (Table S15; Data set S5).

### Pathway evolution: ancestral state reconstruction

**Fig 6:**
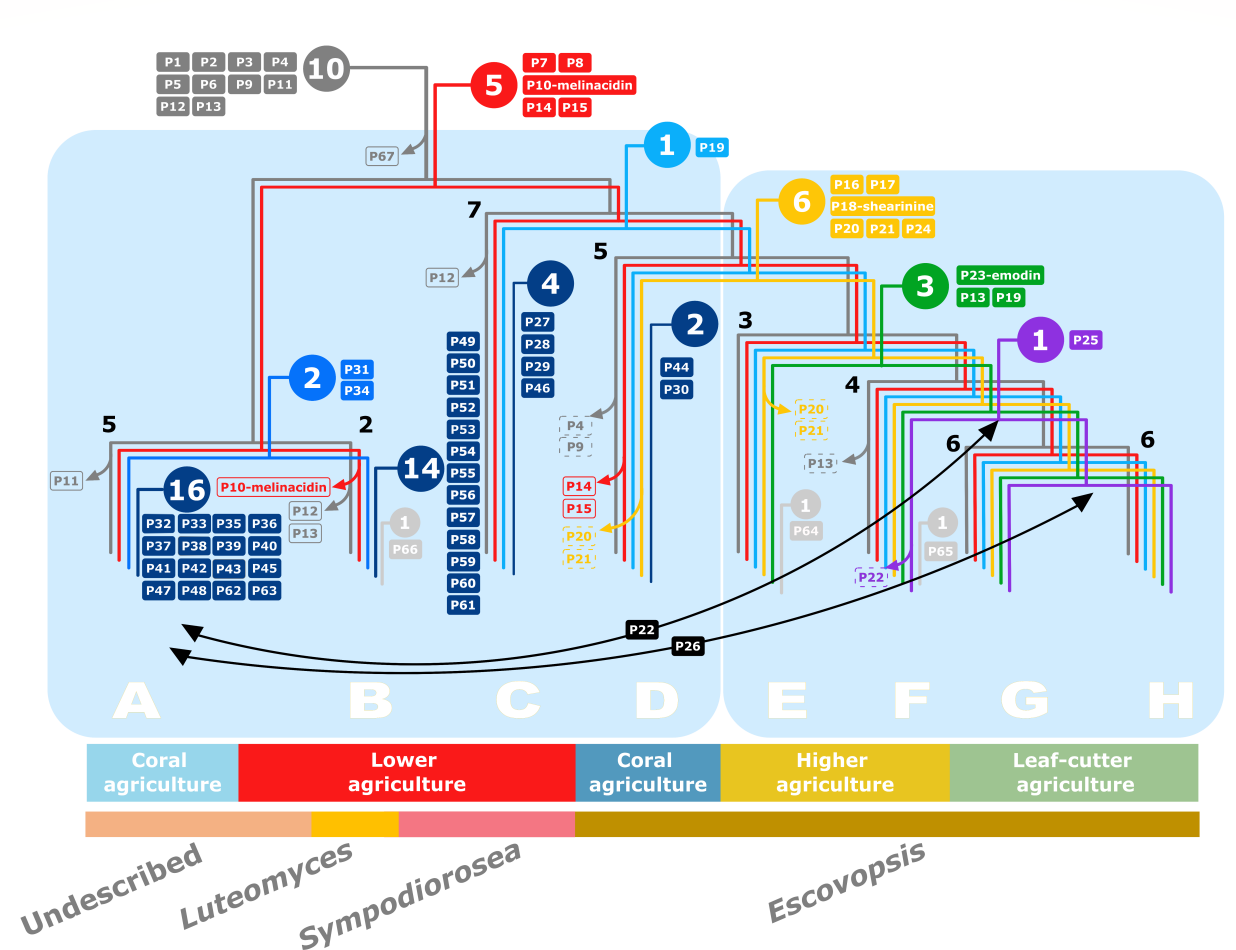
Phylogeny depicting the inferred ancestry of secondary metabolite biosynthetic pathways. A simplified species phylogenomic tree depicts eight major lineages (**A–H**), which correspond to the newly proposed taxonomical divisions. The number of strains in each group is indicated in black adjacent to branch nodes. Circles indicate the number of pathways originating at various points in the species tree, whereas filled boxes indicate pathways next to the point of acquisition. Transparent boxes represent pathway losses in all strains within a clade (continuous outline) or more than 50% of the strains in the clade (dashed outline). Branches are colored according to the following: gray, pathways shared with the sister clade; red, shared with the common ancestor of the genus; indigo, present in the clade comprising an undescribed genus and *Luteomyces*; light blue, present in the monophyetic clade comprising *Sympodiorosea* and *Escovopsis*; yellow; shared between *Escovopsis* strains infecting coral agriculture and general higher agriculture; green, shared by all higher agriculture *Escovopsis*; purple, shared by derived general higher agriculture and leaf-cutter agriculture *Escovopsis*; dark blue, clade-specific pathways; light gray, strain-specific pathways. Black arrows indicate putative horizontal gene transfer events.

To explore the evolutionary history of the symbionts’ biosynthetic pathways relative to their encoding strains, we performed an ancestral state reconstruction analysis. We clustered GCFs into pathways (Ps) based on the assumption that they produce related compounds (see Materials and Methods, Table S17). The 415 GCFs detected in our data set were clustered into many different pathways. Their distribution was overlaid onto a simplified symbiont phylogeny, generated by collapsing certain branches on the species tree, resulting in eight lineages (A–H), which correspond with the newly described genera (A, undescribed genus; B, *Luteomyces*; C, *Sympodiorosea*; and D–H, *Escovopsis*) (Fig. 6). Sixty-seven pathways were present in the symbionts, out of which 56 were unique to this group of symbionts and 11 were shared with other *Hypocreales*. The analysis revealed that 15 pathways were present in the common ancestor of escovopsis symbionts, and 11 of those were shared with the closely related genus *Cladobotryum*. The transition from a non-ant-associated lifestyle to a fungal garden inhabitant correlates with the loss of one pathway (P67), which is involved in the biosynthesis of an uncharacterized PKS and is present in all close relatives but absent in every attine-associated symbiont. Five pathways (P7, P8, P10, P14, and P15) evolved early in the evolutionary history of these fungal symbionts and are present in most strains. However, none of them are ubiquitous, as there have been some clade-specific losses.

The remaining 50 pathways were acquired at various points during the evolution of the group, either through horizontal gene transfer (HGT) or *de novo*. An average of three pathways are acquired with every transition to a new ant agricultural system. However, the transition from strains within the three most ancestral clades (A–C, *Sympodiorosea*, *Luteomyces,* and an undescribed genus) to the most derived super-clade, including clades D–H (*Escovopsis*), correlates with the acquisition of five pathways, including P18, predicted to be responsible for the biosynthesis of shearinine D. This indicates that these pathways are unique to *Escovopsis* spp. Four pathways evolved early in the divergence of *Escovopsis* to infect higher attine agriculture. Interestingly, no pathway is unique to the most derived clade of leaf-cutter ant-associated *Escovopsis*, clades G and H.

Phylogenetic analysis of key biosynthetic genes from each pathway confirms, based on congruence with the species tree, vertical inheritance for most of the pathways following acquisition. However, it also suggests that some pathways may have been exchanged between strains. P22, encoding for a terpenoid, has been transferred between the ancestor of strains exploiting higher attines (ancestor of clades F–H) and the clade comprising an undescribed genus infecting coral agriculture (clade A). Similarly, P26, encoding a PKS, seems to have been shared between the ancestor of strains infecting leaf-cutter agriculture and the most derived clade infecting coral agriculture. In both cases, the direction of the exchange remains unclear. However, once transferred, these pathways have subsequently been vertically inherited by all members of the clades.

The evolution of biosynthetic potential in these symbionts has not only evolved through pathway acquisition, but also through BGC losses. Six pathways have been lost in strains infecting lower attines: three that were already present in the sister clade represented by *Cladobotryum* and *Hypomyces rosellus* (P10, 11, 12, and P13) and three that evolved in the common ancestor of all attine-associated symbiont strains (P10, P14, and P15). P12 appears to have been lost twice, once in clade B (*Luteomyces*) and once in clade C (*Sympodiorosea*). P4 and P9 have also been lost in four and two strains, respectively. Within *Escovopsis* parasitizing higher attine colonies, no pathway has been lost completely. Only three pathways have been lost in some strains: the ancient P13 in clade F, and the more recently evolved P18, putatively encoding for shearinine, in one clade E strain (EPCORN). While the loss of this BGC in EPCORN and its inability to synthesize the resulting compound was already described through both bioinformatic and chemical assays (31), our results suggest it is not a widespread event, given that all the remaining strains still conserve the BGC. A number of pathways (P4, P9, P14, P15, P20, P21) have been lost in *Escovopsis* spp. strains that experienced a host-shift, from association with a *Leucocoprineae* to a *Pterulaceae* cultivar host. It is plausible that these pathway losses represent an adaptation and specialization to exploit a new host. In general, more pathways have been lost in symbionts strains infecting lower attine gardens than those *Escovopsis* spp. attacking the cultivars of higher attines, and those pathways were most often ancient, suggesting that newly acquired BGCs either (i) have not had enough evolutionary time to be selected against or (ii) may be adaptive and thus maintained. These results oppose patterns described in other fungi, where generalist parasites harbor more BGCs than specialist ones (4). In this symbiont group, strains infecting lower attines are thought to be less specialized than those infecting higher attine gardens (22). However, our results suggest that they may be more specialized than previously thought. Furthermore, the colonies of lower attines, consisting of a handful of workers, are smaller than those of higher attines, which consist of millions of workers. Given the insecticidal properties of some BGCs, it is plausible that parasitic strains attacking bigger colonies require a more diverse cocktail of bioactive compounds relative to those infecting smaller colonies in order to prevent clearance by the ants. In fact, studies have demonstrated that the proportion of ant nests harboring fungal contaminants (fungi other than the cultivar) is highest in lower attines (16). However, the proportion of those contaminants made up by *Escovopsis* spp. is highest for higher attines (16). This could be the result of *Escovopsis*’ greater ability to fend off competitors and to inhibit ant-weeding behavior, relative to other symbionts infecting lower attine nests, given their higher content in BGCs. Additionally, symbiont strains in small colonies may encounter less diverse microbial communities compared to those encountered in bigger gardens, and as such may not require as many antibiotic compounds to outcompete other microbes.

Our results suggest that atttine-ant associated symbionts acquired the capacity to synthesize the antimicrobial compound melinacidin IV early in their evolution. It was subsequently lost in lineage B (P10), i.e., *Luteomyces* infecting lower attine gardens. The evolution of the pathway is, however, uncertain. Although we did not detect the presence of the core biosynthetic enzymes in the attine-associated symbionts’ sister clade, consisting of *Cladobotryum* and *Hypomyces* strains, other *Hypocreales*, such as *Acrostalagmus* sp., a rare fungal genus that has been found associated with soil (117), mushrooms (118), and plant material (119), are known to synthesize this metabolite. This suggests that this BGC may have been acquired horizontally. However, while the closely related genus *Trichoderma* has never been described to synthesize this antibiotic, strains within this genus harbor a number of homologous genes to the melinacidin IV BGC, including the backbone enzyme (120). Therefore, alternatively, it is plausible that the pathway responsible for the production of melinacidin IV evolved early within the *Hypocreaceae* family and was lost in the *Cladobotryum-H. rosellus* clade, accumulating enough changes (or requiring fewer genes than previously thought) that we have classified them as different GCFs in our survey.

The inferred ancestry for the pathway predicted to be responsible for shearinine (P18) biosynthesis suggests that it is characteristic of *Escovopsis* spp. While absent from other *Hypocreales*, a BGC encoding for shearinine D has been described for the distantly related fungus *Penicillium janthinellum* (121), suggesting that it may have evolved through HGT in these symbionts. Emodin, encoded by pathway P24, was one of the last BGCs to evolve within *Escovopsis*, appearing in the ancestor of strains parasitizing general higher agriculture and leaf-cutter ants. Our current understanding of escovopsis symbionts’ strain variation and BGC content will undoubtedly improve with further taxon sampling across the symbionts’ phylogeny and will help elucidate the relative contribution of HGT and *de novo* origin to their chemical potential.

The evolutionary transition between lower to higher agriculture in attine ants correlates not only with an increase in colony size (from hundreds to millions of workers) (122) but also with an incipient division of labor between worker ants that culminates with the cast system in leaf-cutter ants (123). The transition from infecting lower to higher attine agriculture gardens coincided with the evolution of a new suit of biosynthetic gene clusters, possibly explaining the increase in complexity required by these symbionts to survive in this environment.

### Conclusion

Microbial symbionts interact with their hosts and competitors through a remarkable array of secondary metabolites and natural products. Here, we highlight the highly streamlined genomic features of attine ant-associated symbionts that are best known as parasites of the ancient agricultural systems. The genomes of *Escovopsis* spp., as well as species from the other symbiont genera, are defined by seven chromosomes, harboring few repetitive sequences. Despite a high degree of metabolic conservation, we observe variation in the symbionts’ potential to produce secondary metabolites. As the phylogenetic distribution of the encoding biosynthetic gene clusters coincides with attine transitions in agricultural systems and cultivar types, we highlight the likely role of these metabolites in mediating adaptation by a group of specialized symbionts. Future efforts will shed light on the mode-of-action and mechanistic basis of these secondary metabolites and how these metabolites relate to the symbionts’ lifestyles and interactions with other members of this ancient agricultural system.

## Data Availability

Raw WGS data is available on GenBank under the BioProject accession PRJNA1059163. Assembled genomes, supplementary figures, and tables, as well as OrthoFinder, AntiSMASH, and BigScape outputs can be found in FigShare under doi: 10.6084/m9.figshare.c.6976881.
